# Blocking β-alanine synthesis triggers widespread perturbations of energy and lipid metabolism in the brain

**DOI:** 10.1016/j.molmet.2026.102383

**Published:** 2026-05-23

**Authors:** Selina Cannon Homaei, Elaheh Mahootchi, Aashish Srivastava, Mahima Sanjay Gomladu, Oda Caspara Krokengen, Anne Baumann, Jan Haavik

**Affiliations:** 1 Department of Biomedicine, University of Bergen, Bergen, Norway; 2 Genome Core-Facility, Clinical Laboratory (K2), Haukeland University Hospital, University of Bergen, Bergen, Norway; 3 Division of Psychiatry, Haukeland University Hospital, Bergen, Norway

**Keywords:** Glutamate decarboxylase-like 1 (GADL1), β-alanine metabolism, Carnosine, Fatty acid synthesis, Energy homeostasis, Multi-omics

## Abstract

**Background:**

Glutamate decarboxylase-like 1 (GADL1) decarboxylates aspartic acid to β-alanine in several mammalian tissues, particularly in the brain and skeletal muscle. β-alanine is a precursor to the antioxidant and osmoregulatory dipeptide carnosine (β-alanyl-l-histidine), as well as pantothenic acid and coenzyme A. Deletion of GADL1 reduces carnosine and anserine levels in multiple tissues, but the consequences for brain metabolism remain unclear. This study aimed to explore sex-specific metabolic and cellular effects of GADL1 and β-alanine depletion in different areas of the brain.

**Methods and results:**

We conducted a metabolomic screening of seven mouse tissues, followed by a detailed transcriptomic, proteomic, and metabolomic analysis of cerebrum, cerebellum, and olfactory bulb tissues from male and female GADL1 knockout and wild-type mice to explore sex-, age-, and region-specific molecular alterations. Loss of GADL1 induced distinct, sex-dependent metabolic responses across brain regions. Metabolomic data showed increased oxidative stress and possible synaptic remodeling in the cerebrum of mature females, whereas males exhibited massive lipid accumulation in multiple tissues. A similar pattern appeared in the developing olfactory bulb, where both sexes displayed lipid accumulation, but only males showed signs of inflammatory activation and altered energy metabolism, as supported by transcriptomic and proteomic analyses.

**Conclusions:**

GADL1 loss and consequent β-alanine depletion trigger widespread metabolic remodeling in brain tissue. Even modest β-alanine reduction leads to region, age, and sex-specific perturbations of energy metabolism and cellular homeostasis. These findings highlight the multifaceted biochemical roles of β-alanine and suggest that its physiological and therapeutic effects may differ by tissue, sex, and developmental stage.

## Highlights

• Multi-omics profiling reveals sex-specific metabolic responses to GADL1 loss.
• Female mice exhibit oxidative stress and synaptic remodeling signatures.
• Male mice show lipid accumulation and inflammation-related alterations.
• β-alanine depletion causes distinct metabolic rewiring.

## 1 Introduction

β-alanine is a naturally occurring non-proteogenic amino acid and a widely used ergogenic supplement in sports nutrition [1]. It is synthesized in animal tissues via multiple metabolic pathways or obtained through the diet and serves as the rate-limiting precursor of the dipeptide carnosine (β-alanyl-l-histidine). Although the biological functions of β-alanine are not fully understood, dietary supplementation with this amino acid consistently elevates skeletal muscle carnosine levels, thereby enhancing intramuscular pH buffering capacity during high-intensity exercise [2]. Beyond its role in exercise physiology, carnosine also functions as an antioxidant and osmotically active dipeptide that has been proposed as a biomarker of energy homeostasis [3].

Carnosine and related dipeptides exert a wide range of biological effects, including antioxidation, metal chelation, anti-glycation, calcium (Ca^2+^) regulation, and modulation of neurotransmission [[4], [5], [6]]. These multifunctional properties make carnosine an intriguing therapeutic target in metabolic, age-related, and degenerative diseases. Recent studies have further demonstrated that carnosine production in malignant cells promotes tumor survival under acid stress, suggesting a role in cancer biology [7]. Similarly, carnosine treatment has been reported to alleviate oxidative stress and neuroinflammation following ischemic stroke [8]. Endogenous carnosine levels show pronounced sexual dimorphism in both humans and rodents [9,10].

Glutamate decarboxylase-like 1 (GADL1) is a pyridoxal 5′-phosphate (PLP)-dependent enzyme that belongs to a family of structurally related decarboxylases. *In vitro*, the enzyme shows a preference for the conversion of l-aspartic acid to β-alanine, but it can also decarboxylate other structurally similar substrates, such as cysteine sulfinic acid [[11], [12], [13]]. Among mammalian tissues, GADL1 is primarily expressed in the brain and skeletal muscle tissues (BrainSpan, https://www.brainspan.org/static/home; Human Brain Atlas, https://human.brain-map.org/microarray/gene/show/109758), with peak expression levels observed during embryonic development [13]. In the brain and spinal cord, GADL1 is mainly expressed in oligodendrocytes and is upregulated in response to oxidative stress [13,14].

In a longitudinal follow-up study, boys had a 10–24 % increase in carnosine levels from pre-to post-puberty, while a puberty-linked increase in girls was not detected, indicating that the sexual dimorphism observed in adult muscle tissue (20–29 % higher in males) could be related to testosterone production [15,16]. During high-intensity exercise, skeletal muscle adapts by shifting energy metabolism towards anaerobic glycolysis, and elevated carnosine levels help buffer intracellular pH, thereby improving athletic performance [9,17]. However, because carnosine is rapidly hydrolyzed upon ingestion, oral supplementation of its precursor, β-alanine, has become the preferred strategy among athletes [4]. Importantly, the regulation of β-alanine and carnosine levels is closely linked to GADL1 activity, highlighting the need to investigate how disruption of this pathway impacts metabolism and energy homeostasis.

Beyond its role in carnosine-related peptides, β-alanine may also have other physiological functions, including acting as a small neurotransmitter/neuromodulator [18] and stimulating oxidative metabolism and mitochondrial biogenesis [19]; however, these functions are not fully understood. Its synthesis and transport are tightly regulated through multiple pathways that vary by species and tissue [[20], [21], [22], [23]]. In addition to its *de novo* biosynthesis through pyrimidine or vitamin B5 catabolism and its dietary supply as a free amino acid or peptide derivative, pure β-alanine has recently become popular as a dietary supplement [24].

Research on GADL1 remains limited, and its biochemical roles beyond β-alanine synthesis are largely unexplored. We recently reported the first *Gadl1* knockout (KO) mouse model. Deletion of *Gadl1* led to a tissue-specific depletion of β-alanine, carnosine, N-acetyl-carnosine, and anserine in skeletal muscle, olfactory bulb (OB), and cerebrum tissue, whereas these metabolite levels were largely unchanged in liver, kidney, cerebellum, and heart. Moreover, the *Gadl1* KO mice exhibited elevated markers of oxidative stress and altered anxiety-like behavior [11].

Building on these observations, we aimed to investigate the sex- and tissue-specific metabolic, proteomic, and transcriptomic consequences of GADL1 deletion, as well as the broader biological effects of β-alanine depletion using the *Gadl1* KO mouse model. We hypothesized that GADL1 inactivation and the resulting reduction in β-alanine and carnosine would trigger a metabolic shift impacting energy metabolism. Given GADL1's tissue-specific expression and the known sexual dimorphism in carnosine levels, we further wanted to explore potential sex-specific effects of GADL1 loss.

To test these hypotheses, we conducted an untargeted multi-omics study in *Gadl1* KO and wild-type (WT) mice using metabolomics (LC-MS), proteomics (LC-MS), and transcriptomics (mRNA sequencing), focusing primarily on the brain regions OB and cerebrum. These brain regions were selected because they showed the most significant depletion of β-alanine and carnosine peptides following GADL1 deletion [11]. Here, we report that GADL1 deficiency also disrupts energy metabolism through a complex network of pathways, including mitochondrial function, neuromodulation, and fatty acid transport.

## 2 Materials and methods

### 2.1 Animal studies and tissue collection

All animal experiments were approved by the Norwegian Food Safety Authority (FOTS id 17443) and designed following the ARRIVE guidelines. The constitutive *Gadl1* KO model was generated in C57BL/6N mice at genOway (Lyon, France). Details on generation, breeding, genotyping, validation, and diet have been reported previously [11]. Mice were group-housed under standardized conditions (21 ± 1 °C; 12:12 h light/dark cycle). Animals were anesthetized with urethane (250 mg/mL, 1.4–1.8 mg/kg, i.p.) and perfused with 0.02 % heparin-saline prior to organ collection. Tissues were flash-frozen in liquid nitrogen and stored at −80 °C until analysis.

### 2.2 Metabolic studies (liquid chromatography-mass spectrometry)

We analyzed 172 tissue samples collected from 41 mice using liquid chromatography-mass spectrometry (LC-MS) at Metabolon Inc. (Durham, NC, USA). Between 541 and 720 known metabolites were detected and quantified per sample. Sample processing and LC-MS analysis followed Metabolon's established protocols, as previously described [11]. Normalized compound areas were rescaled so that the median equaled 1. Metabolomic alterations were assessed in the OB, cerebrum, cerebellum, kidney, liver, skeletal muscle, whole blood, and plasma (see Supplementary Table a1A and a1B). Data were filtered by relative standard deviation (RSD, 25 %) and normalized using Pareto scaling. Exploratory analyses were performed in MetaboAnalyst 6.0 and RStudio (2025.05.1 + 513). Partial least squares-discriminant analysis (PLS-DA) identified the top 15 metabolites ranked by variable importance in projection (VIP) score. Hierarchical clustering heatmaps displayed the top 25 metabolites using Euclidean distance measure and Ward clustering. The robustness of the clustering was validated by calculating average silhouette widths in R (‘cluster’), based on the features included in the heatmaps, ensuring the quantitative metrics directly reflect the presented visual clusters. Metabolite Set Enrichment Analysis (MSEA) was performed using HDMB IDs mapped to KEGG pathways. Glycerolipid (mono- and diacylglycerol) levels were analyzed by random-effects meta-analyses using Metafor [25].

### 2.3 Proteomics

#### 2.3.1 Sample digestion

Cerebrum tissue (20 mg) was collected from male (WT, n = 6; KO, n = 6) and female (WT, n = 6; KO, n = 6) *Gadl1* mice for proteomics analysis. Samples were homogenized and lysed in 200 μL 4 % SDS/0.1 M Tris–HCl (pH 7.6) before rod sonication (3 × 30 s, 30 % amplitude, 30 s rest). Lysates were denatured at 95 °C for 7 min and centrifuged at 13,000 rpm for 10 min. A 150 μL aliquot was frozen (−20 °C) for proteomics preparation, while the remainder was used for BCA protein quantification. Protein digestion followed the SP3 protocol described in Szigetvari et al. [26]. Briefly, 250 μg of beads (1:1 of Sera-Mag SpeedBeads, 50 mg/mL (GE Healthcare, cat. no. 45152105050250) and Sera-Mag SpeedBeads, 50 mg/mL (GE Healthcare, cat. no. 65152105050250) were added per sample. Protein binding was induced by adding ethanol to a final concentration of 70 %, followed by incubation (7 min, 24 °C, 1000 rpm). Beads were magnetized, and unbound material was removed. After two washes with 80 % ethanol, bead-bound proteins were digested with trypsin (protein: trypsin ratio = 50:1) in 100 mM ammonium bicarbonate/1 mM CaCl_2_ at 37 °C for 16 h. Peptides were eluted, beads washed once with 0.5 M NaCl, and eluates combined. Samples were desalted using Oasis HLB 96-well μElution plates and resuspended in 2 % acetonitrile, 0.5 % formic acid for LC-MS analysis (DIA mode).

#### 2.3.2 DIA (data-independent-acquisition) method (95 min run)

Proteomic analysis was performed at PROBE using nanoLC-Orbitrap Eclipse mass spectrometry (Thermo Scientific, Bremen, Germany). Approximately 0.6 μg protein as tryptic peptides (in 2 % acetonitrile (ACN), 0.5 % formic acid (FA)) was injected into an Ultimate 3000 RSLC system (Thermo Scientific, Sunnyvale, California, USA) connected online to an Orbitrap Eclipse equipped with EASY-spray nano-electrospray ion source (Thermo Scientific).

**Trapping and desalting:** Samples were loaded onto a pre-column (Acclaim PepMap 100, 2 cm × 75 μm ID nanoViper, 3 μm C18) at a 5 μL/min flow rate for 5 min with 0.1 % trifluoroacetic acid.

**LC separation (95 min):** Peptides were separated at 300 nL/min on a 15 cm Aurora Elite XT column (75 μm ID, C18, 40 °C) using a biphasic acetonitrile gradient from two nanoflow UPLC pumps. Solvent A was 0.1 % FA in water, and solvent B was 100 % ACN. The gradient was: 5 % B (5 min, trapping), 5–7 % B (30 s), 7–35 % B (62.5 min), and 35–85 % B (2 min). Very hydrophobic peptides were eluted isocratically at 85 % B for 2 min. A three-cycle seesaw gradient (85–5 % B over 2 min, 5 % B at 2 min, 5–85 % within 1 min, 85 % B for 2 min) was then applied, followed by equilibration at 5 % B for 7 min.

**DIA (data-independent-acquisition) with FAIMS:** Eluted peptides were analyzed with FAIMS enabled at a compensation voltage (CV) of −45 V. The mass spectrometer operated in DIA-mode to automatically switch between one full MS scan and MS/MS acquisition of 42 mass segments (cycle time = 3 s). Instrument control was through Orbitrap Eclipse Tune 3.5 and Xcalibur 4.5. MS spectra were acquired in the scan range 400–900 *m*/*z* at 120,000 resolution (200 *m*/*z*), with automatic gain control (AGC) target of 4e5 and a maximum injection time (IT) set to 50 ms. Using an isolation window of 12 Da (first window 399.4–412.4 *m*/*z* and the last from 891.6 to 904.6 *m*/*z*), all ions in the *m*/*z* window were sequentially isolated to a target value (AGC) of 4e5 and a maximum IT of 22 ms in the C-trap before undergoing higher-energy collision dissociation. Fragmentation was performed with a normalized collision energy of 30 %, and fragments were detected in the Orbitrap at a resolution of 15,000 at m/z 200, with scan range fixed at *m*/*z* 145–1450 *m*/*z*. Lock-mass internal calibration was not enabled.

**Ion source parameters:** Ion spray voltage was set to 1900 V, no sheath and auxiliary gas flow (0), and capillary temperature was maintained at 275 °C.

#### 2.3.3 Data analysis with spectronaut 19

Raw DIA files were processed in Spectronaut (direct DIA mode). Trypsin was specified as the digestion enzyme, with carbamidomethylation set as a fixed modification and methionine oxidation and N-terminal acetylation as variable modifications. Quantification was performed at the MS2 level without data imputation. Searches were performed against the SwissProt mouse protein database (downloaded March 13th, 2025) combined with a common contamination database (March 2022).

### 2.4 Transcriptional studies, mRNA sequencing

RNA was purified from *Gadl1* KO and WT brain regions, OB and cerebrum, using a RNeasy fibrous tissue kit (Qiagen, #74704). A total of 48 samples were prepared, matched by tissue, sex, and genotype (see Supplementary Table a2). All mice were mature adults, aged 34–44 weeks. RNA sequencing was performed on a NovaSeq 6000 platform using the S1 Reagent Kit v1.5 (200 cycles). Raw reads were aligned to the mouse reference genome GRCm38 (mm10) with GENCODE M13 annotation (GENCODE, Mouse release M13) using HISAT2 [27]. The resulting BAM files were used for gene-level quantification with featureCounts [28]. Normalization and differential gene expression (DGE) analyses were performed with DESeq2 [29]. Between 16,429 and 23,522 transcripts were detected per group. Differentially expressed genes were defined by *p* < 0.05, absolute fold change ≥1, and baseMean ≥ 10. Volcano plots were generated with EnhancedVolcano [30]. Functional enrichment analyses were performed with clusterProfiler [31] using Gene Ontology (GO) [[32], [33], [34]] and KEGG pathway database [[35], [36], [37]].

### 2.5 Statistical analysis

Statistical analyses were performed in GraphPad Prism 10 (Version 10.4.1 (532)) or RStudio (2025.05.1 + 513). Significance was defined as *p* < 0.05 and absolute log2 fold change ≥1, unless otherwise specified. Group comparisons were conducted using Student's *t*-test. Proteomics data were processed in Perseus. Principal component analysis (PCA) was performed to assess global variance across tissues and omics layers (Supplementary Fig. a1).

## 3 Results

### 3.1 Loss of GADL1 induces sex-dependent metabolic changes across brain regions

**Figure 1:**
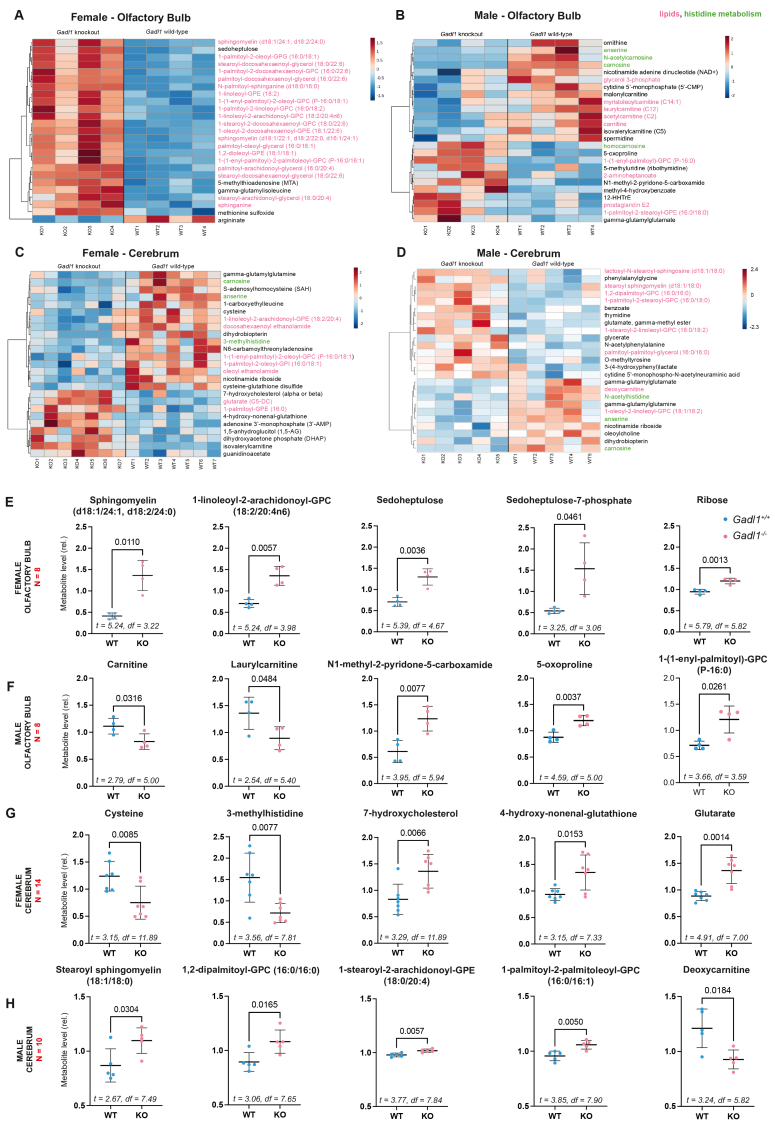
**GADL1 depletion causes sex-specific metabolic alterations**. Untargeted metabolomics was performed on the cerebrum and olfactory bulb of female and male *Gadl1* mice. (A–D) Hierarchical clustering heatmaps of the top 25 metabolites generated using Euclidean distance measure and Ward clustering for (A) female and (B) male olfactory bulb, (C) female and (D) male cerebrum. Lipids are marked in pink; histidine metabolites are marked green. Quantitative validation of the clustering was performed using silhouette analysis. The average silhouette widths for each panel are: (A) 0.71, (B) 0.33, (C) 0.40, and (D) 0.38, indicating moderate to strong separation between the groups. (E–H) Representative selection of significantly altered metabolites for (E) female and (F) male olfactory bulb, (G) female and (H) male cerebrum. Analyses were performed in MetaboAnalyst (v.6.0), RStudio (2026.04.0 + 526), and GraphPad Prism (v.10.4.1, 532).

An untargeted metabolomics analysis using LC-MS on three brain regions (OB, cerebrum, and cerebellum) was performed in female and male *Gadl1*^*−/−*^ and *Gadl1*^+/+^ mice. The results confirmed that the deletion of *Gadl1* triggers a dysregulation of the β-alanine pathway, with consistent and widespread depletion of the histidine-containing dipeptides (HCDs) carnosine, N-acetylcarnosine, and anserine (10–90 % reduction across different tissues), as reported in Mahootchi et al. [11]. Sex- and tissue-specific hierarchical clustering was conducted to visualize inherent metabolic relationships, showing robust grouping of samples by genotype, driven by distinct metabolic signatures (Figure 1). To validate the discriminatory power of the top 25 metabolites visualized in each heatmap, we calculated average silhouette widths. The scores were (Fig. 1A) 0.71 (Fig. 1B), 0.38 (Fig. 1C), 0.40, and (Fig. 1D) 0.33, confirming a moderate to robust cluster structure for the selected metabolic signatures. In accordance with the reported tissue distribution of HCDs in mice, rats, and humans [38], the strongest effects were observed in the OB and cerebrum, while only modest changes were detected in the cerebellum (Supplementary Fig. a2). Thus, subsequent analyses focused primarily on OB and cerebrum tissues. Males also displayed greater variability in metabolite levels between individuals, suggesting a more heterogeneous metabolic response to GADL1 loss.

In the OB, the most prominent metabolic changes in females included lipid accumulation and reduced antioxidant capacity (Fig. 1A). The results show elevated levels of several sphingolipids and glycerolipids (Fig. 1E), including sphingomyelin (d18:1/24:1, d18:2/24:0) and 1-linoleoyl-2-arachidonoyl-GPC (18:2/20:4n6). Elevation of sedoheptulose, sedoheptulose-7-phosphate, and ribose indicated activation of the pentose phosphate pathway, suggesting a possible rerouting from glycolysis. In contrast, males exhibited depletion of carnitine and laurylcarnitine, alongside a significant accumulation of 5-oxoproline and N1-methyl-2-pyridone-5-carboxamide (Fig. 1B,F). These findings suggest a sex-specific impairment of the carnitine shuttle and altered glutathione metabolism, primarily shown as altered energy metabolism and mitochondrial stress response in males, rather than the lipid accumulation seen in females.

In the cerebrum, the results indicate depletion of metabolites associated with oxidative stress and energy dysregulation. Depletion of protective metabolites (HCDs, cysteine) and elevation of oxidative damage markers (7-hydroxycholesterol and 4-hydroxy-nonenal-glutathione) distinguished the genotypes in the female cerebrum (Fig. 1C). Signs of altered mitochondrial metabolism and energy production were also evident, reflected in changes in isovalerylcarnitine, glutarate, and nicotinamide riboside. Additionally, significant alterations were detected in key lipids and amino acids, including glycerophospholipids, docosahexaenoyl ethanolamide, and γ-glutamylglutamine (Fig. 1G).

While the male cerebrum (Fig. 1D) exhibited similar functional deficits, GADL1 depletion affected distinct metabolic pathways. For instance, accumulation of saturated glycerolipids and sphingolipids, alongside depleted levels of deoxycarnitine, indicated a lipid profile shift similar to the observed alterations in the female OB (Fig. 1H). Together, these observations suggest that *Gadl1*^*−/−*^ is associated with widespread metabolic alterations, with sex- and tissue-specific differences in energy metabolism and antioxidant defense.

### 3.2 Gadl1 deletion alters lipid metabolism across the brain and peripheral tissues

To evaluate the systemic impact of *Gadl1* deletion on lipid metabolism, a comparison of metabolite levels in liver, kidney, skeletal muscle, plasma, and brain tissues from *Gadl1*^*−/−*^ *and Gadl1*^+/+^ mice was performed, focusing on mono- and diacylglycerols. Random-effects meta-analyses revealed significant alterations across multiple datasets (Supplementary Table a3). Monoacylglycerol levels were significantly altered (*p* < 0.05) in the skeletal muscle and cerebrum of both sexes, and additionally in the male liver, plasma, and cerebellum. Diacylglycerol levels were strongly elevated in skeletal muscle and liver across sexes, as well as in the female OB and male kidney and cerebellum. These results highlight tissue-specific vulnerability and reveal sex-dependent differences in the metabolic stress response of *Gadl1*-deficient mice.

**Figure 2:**
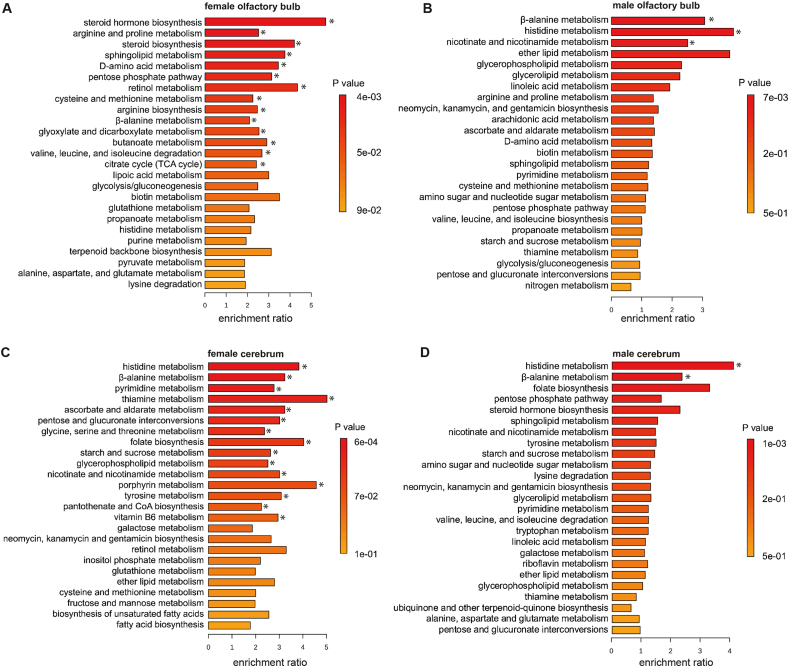
**Affected metabolic pathways in GADL1 knockout mice**. MSEA was performed on LC-MS data from (A) female and (B) male olfactory bulb, (C) female and (D) male cerebrum. Female mice exhibited increased pathway impact; however, β-alanine and histidine metabolism were consequently enriched. Significant pathways (p < 0.05) are marked with an asterisk (∗).

MSEA was then performed to identify the metabolic pathways most affected by GADL1 deletion in the cerebrum and OB (Fig. 2A–D). Female mice exhibited more pronounced pathway alterations than males, with 18 vs 4 enriched pathways in the OB and 15 vs 2 in the cerebrum (*p* < 0.05). As expected, β-alanine and histidine metabolism were consistently enriched across all datasets. In the female OB, enriched pathways included sphingolipid metabolism (*p* = 4.23e^−4^) and the pentose phosphate pathway (*p* = 0.021), whereas males showed enrichment only in nicotinate and nicotinamide metabolism (*p* = 0.012) and ether lipid metabolism (*p* = 0.032). In the female cerebrum, pyrimidine metabolism (*p* = 0.005) and glycerophospholipid metabolism (*p* = 0.0124) were among the most affected pathways. No additional pathways were significantly enriched in males. As also shown in Figure 1, the limited pathway enrichment in males likely reflects greater biological variability, which may mask pathway-level changes. Overall, these analyses indicate that GADL1 triggers sex- and tissue-specific disruptions in lipid metabolism, energy pathways, and redox balance, with females showing a more comprehensive and coordinated metabolic response.

### 3.3 Sex-specific proteomic changes in the Gadl1-deficient cerebrum

**Figure 3:**
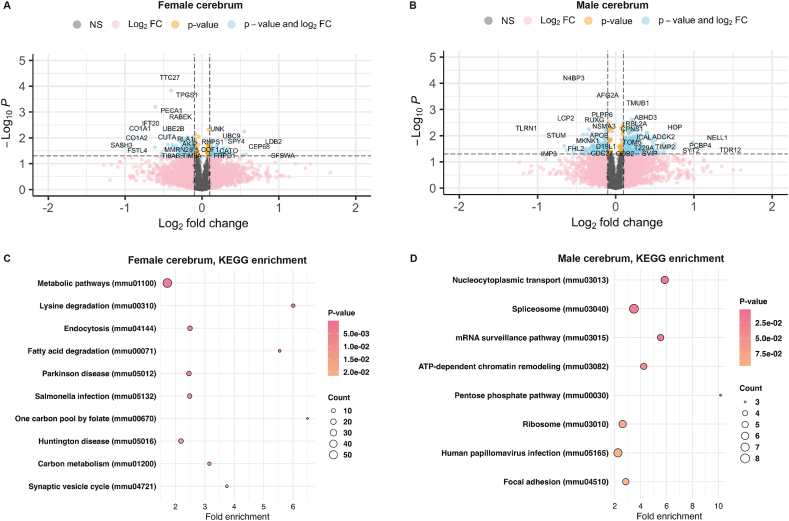
**Proteomic profiling reveals metabolic, oxidative, and neurotransmission alterations in *Gadl1* knockout mice**. We performed mass-spectrometry proteomic analysis of the male and female adult cerebrum. (A–B) Volcano plots of significantly (*p* < 0.05, absolute log_2_ fold change ≥0.1) altered proteins in (A) female and (B) male cerebrum. (C–D) Functional KEGG enrichment analysis in (C) female and (D) male cerebrum.

Untargeted mass-spectrometry proteomics was performed on cerebrum tissue from male and female *Gadl1*^−/−^ mice to characterize sex-dependent alterations (Figure 3). A total of 7726 proteins were detected. Given that *Gadl1*^*−/−*^ mice exhibit a mild developmental and behavioral phenotype [11], a lenient threshold was applied to detect possible changes (*p* < 0.05, absolute log2 fold change ≥0.1). In these exploratory analyses, more significant protein alterations were detected in females compared to males (326 vs 159), mirroring the broader metabolic changes detected in females via metabolomics. This suggests that GADL1 deletion exerts a stronger systemic impact in females.

In the female cerebrum (Fig. 3A), proteomic alterations revealed signs of altered synaptic function and redox homeostasis. This pattern included upregulation of synaptotagmin 2 (SYT2), a key calcium sensor in neurotransmitter release, and neural EGF-like 1 (NELL1), suggesting neuronal remodeling. Increased levels of peroxisomal sarcosine oxidase (SOX) pointed to changes in amino acid oxidative metabolism. Conversely, downregulation of talin rod domain-containing protein 1 (TLRN1) and protein stum homolog (STUM) suggests a possible shift in synaptic architecture and excitability. A reduction in the immune adaptor lymphocyte cytosolic protein (LCP2) may imply altered neuroimmune signaling and oxidative stress regulation. Further, several proteins involved in fatty acid metabolism (ACBG1, AL7A1, ECI1, HCDH, ECHB, and GCDH) were modestly upregulated, indicating subtle metabolic remodeling. Notably, we also observed a 14 % increase in expression of cysteine sulfinic acid decarboxylase (CSAD) (*p* = 0.02), an enzyme primarily involved in taurine biosynthesis but with partially overlapping substrate specificity with GADL1 [13,39], suggesting coordinated regulation of these related enzymes.

In the male cerebrum (Fig. 3B), the results pointed to a distinct stress response involving cellular organization and calcium regulation. For example, the *Gadl1*^−/−^ mice showed reduced expression of regucalcin (RGN), a key calcium regulator, as well as S-adenosylmethionine and SH3 domain-containing protein 3 (SASH3), a potential lymphocyte signaling adaptor. In contrast, LIM domain-binding protein 2 (LDB2), involved in transcription and chromatin organization, was elevated, suggesting reorganization of transcriptional and signaling networks. Collectively, these results reveal a female-specific remodeling of synaptic and redox processes and a male-specific shift in calcium and nuclear organization, indicative of divergent strategies of stress adaptation. This distinction was further reflected in pathway-level enrichment analyses.

Functional pathway enrichment (*p* < 0.05) in the female cerebrum (Fig. 3C) highlighted altered fatty acid metabolism (mmu00071) and one-carbon pool by folate (mmu00670), reflecting compensatory shifts in lipid turnover and redox balance under metabolic stress. In addition, enrichment of the synaptic vesicle cycle (mmu04721) and endocytosis (mmu04144) connected metabolic dysregulation to altered neurotransmission and vesicular trafficking. In the male cerebrum (Fig. 3D), enrichment of nucleocytoplasmic transport (mmu03013) pointed to an alternative compensatory mechanism affecting cellular organization. The pentose phosphate pathway (mmu0030) was also enriched, consistent with the MSEA results. Together, the proteomic findings demonstrate that GADL1 deletion drives sex-specific remodeling of cerebral metabolism, neurotransmission, and stress responses. This is consistent with metabolomics, linking β-alanine metabolism to broader effects on neuronal function and homeostasis.

### 3.4 Transcriptomics reveal neuroendocrine and metabolic pathway alterations in GADL1-deficient brain

**Figure 4:**
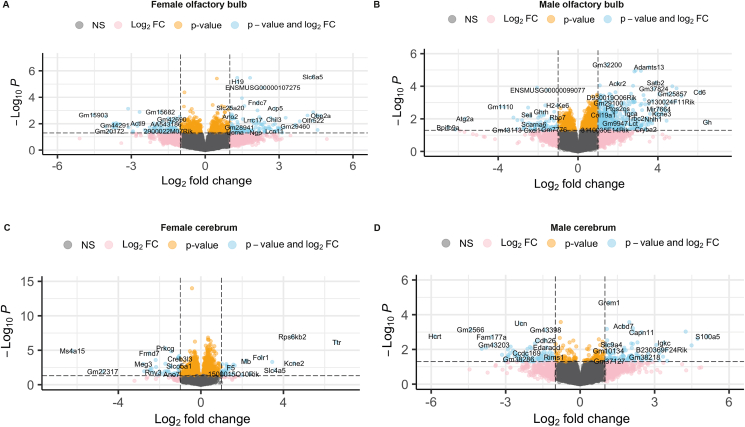
**Sex-stratified transcriptional alterations in GADL1 knockout mice**. mRNA sequencing on 48 samples harvested from the Gadl1 mice. (A–D) Volcano plots of differentially expressed genes in (A) female and (B) male olfactory bulb, (C) female and (D) male cerebrum.

mRNA sequencing was conducted to evaluate the impact of *Gadl1* deletion on gene expression in the cerebrum and OB of male and female mice. Across datasets, 16,429–23,522 transcripts were detected, of which 14–260 genes were significantly altered between WT and KO animals (p < 0.05, |log2FC| ≥ 1, baseMean ≥10), demonstrating pronounced region- and sex-specific transcriptional effects (Fig. 4A–D). Only a small subset of genes was consistently affected across groups, underscoring the selective influence of GADL1 loss. In the cerebrum, *Capn11* (a protease implicated in spermatogenesis and neurological diseases), *Slc23a3*, and *Rps6kb2* (a component of the mTOR signaling pathway) were upregulated, with *Capn11* also increased in the male OB. Although *Slc23a3* is formally classified as an orphan member of the vitamin C transporter family, recent findings suggest a role in hypoxanthine transport [40].

In the OB, consistent upregulation of *Atp2c2*, *Srp45a*, *Tmem232*, and *Kctd16* indicated coordinated changes in ion homeostasis, protein trafficking, and cellular signaling. *Atp2c2* supports neuronal ion balance, *Srp45a* mediates ER-directed protein targeting, ensuring proper protein trafficking, and *Tmem232* is linked to inflammatory signaling, suggesting coordinated adaptations in ion regulation and cellular stress responses. Wu et al. [41] showed that GADL1 overexpression reduced KCTD12 expression in SH-SY5Y neuroblastoma cells, whereas Mahootchi et al. [11] did not observe this effect in mouse tissue. In line with Mahootchi et al., *Kctd12* expression remained unchanged, whereas *Kctd16* (a GABA-B receptor subunit) was increased 2.2-fold (*p* = 0.0071), indicating altered inhibitory neurotransmission. Additionally, *Gabbr2*, encoding the GABA-B receptor 2 subunit, was upregulated 2.3-fold in the male OB (*p* = 0.0048).

Sex-specific analyses revealed distinct transcriptional patterns. In the female OB, increased *Sec14l3* and *Acp5* together with decreased *Txnrd2* and *Npas4* suggested metabolic and synaptic remodeling (Fig. 4A). In males, elevated *Gh* and *Cd6* indicated endocrine and immune modulation, whereas reduced *Coro1b* and *C3* implied cytoskeletal and complement alterations (Fig. 4B). In the cerebrum, female mice showed reduced expression of *Ms4a15*, *Kcne2*, *Meg3*, and *Frmd7*, consistent with altered membrane transport and neuronal excitability (Fig. 4C). Male cerebrum samples displayed marked upregulation of *S100a5* (35-fold), *Igkc* (10-fold) and *Jchain* (4-fold), indicating calcium signaling changes and immune activation (Fig. 4D). Additionally, downregulation of the hypothalamic neuropeptides *Hcrt*, *Pmch*, and *Avp* suggested disruptions in energy balance and stress-response regulation.

Pathway enrichment analysis showed convergence of differentially expressed genes with broader omics signatures (Supplementary Fig. a3), with region-specific effects confirmed by integrative network analysis (Supplementary Fig. a4). The OB exhibited coordinated transcriptomic-metabolomic responses with genotype-dependent clustering, whereas cerebrum changes were more modest, particularly at the proteomic level, where only small shifts in protein abundance were detected. The female cerebrum showed the highest number of enriched pathways, while none were detected in the female OB. Neuroactive ligand–receptor interaction (mmu04081) was enriched in female cerebrum (*p-adj* = 0.007), male cerebrum (*p-adj* = 0.037), and male OB (*p-adj* = 8.8e^−05^), indicating widespread neurotransmitter dysregulation. In females, additional enrichment of dopaminergic synapse (mmu04728, *p-adj* = 0.024), amphetamine addiction (mmu05031, *p-adj* = 0.007), and cocaine addiction (mmu05030, *p-adj* = 0.018) further supported synaptic alterations. Hormone signaling (mmu04080) was enriched in both male cerebrum (*p-adj* = 0.037) and male OB (*p-adj* = 0.006), suggesting endocrine adaptations to GADL1-related metabolic perturbation. Despite limited overlap between significant features, the transcriptional changes in *Gadl1*^*−/−*^ mice aligned with proteomic and metabolomic signatures of altered energy metabolism, neurotransmission, and neuroimmune signaling.

## 4 Discussion

GADL1 is a PLP-dependent enzyme primarily expressed in the brain and skeletal muscle, where it decarboxylates aspartic acid to β-alanine and may also act on structurally similar substrates. Elimination of GADL1 causes a tissue-specific decrease in β-alanine and HCDs, including carnosine, N-acetyl-carnosine, and anserine, and is accompanied by upregulation of other histidine peptides, such as homocarnosine [22]. However, the broader biological functions of GADL1 remain incompletely defined. Here, we used a multi-omics approach to investigate sex-specific secondary metabolic and cellular effects of GADL1 and β-alanine depletion in the mouse brain.

Across metabolomic, proteomic, and transcriptomic analyses, female *Gadl1* KO mice exhibited more altered features than males (Supplementary Fig. a5). This may reflect sex-specific hormonal regulation, as estrogen is known to regulate mitochondrial metabolism [42]. Studies in ovariectomized rats (a postmenopausal model) show that supplementation with histidine, carnosine, cysteine, and serine can mitigate estrogen deficiency by reducing inflammation and improving metabolic resilience [43]. Together, these observations suggest that hormonal context may amplify or buffer the effects of GADL1 loss and β-alanine depletion in females. While these findings suggest a sex-dependent response to GADL1 loss, the sample size was not sufficient to robustly assess sex effects, and these findings should therefore be considered exploratory.

Male mice displayed a narrower but distinct pattern of molecular alterations. In the OB, GADL1 loss reduced carnitine levels, and several medium-to long-chain acylcarnitines showed a downward trend toward significance, suggesting altered substrate availability for mitochondrial fatty acid oxidation [44]. However, the reduced acylcarnitine levels can reflect multiple metabolic alterations, including changes in fatty acid influx, incomplete β-oxidation, or altered carnitine transport, and therefore do not provide direct evidence of impaired mitochondrial function [45]. Nevertheless, altered acylcarnitine profiles have been linked to impaired metabolic and neuroprotective functions in the brain [46]. Notably, reduced acylcarnitines have also been reported in major depressive disorder, schizophrenia, bipolar disorder, and autism spectrum disorder [[47], [48], [49]], linking the observed changes in males to pathways relevant for psychiatric disease. Interestingly, human *GADL1* variants have been associated with response to lithium therapy in bipolar disorder [50]. At the neurotransmission level, GADL1 loss redirected histidine-dipeptide metabolism toward homocarnosine, a GABA-histidine dipeptide that may serve as a localized GABA reservoir. Elevated homocarnosine, combined with transcriptional upregulation of *Gabbr2* and *Kctd16*, could enhance GABA-B receptor signaling [51]. Recent studies have linked GABBR2 overexpression to intracellular Ca^2+^ concentration and reactive oxygen species production, hallmarks of early Alzheimer's disease progression [52]. Despite these alterations, overall omics shifts remained moderate, suggesting that system-level adaptations help maintain metabolic homeostasis, consistent with the mild behavioral phenotype. These adaptations appear to be shaped by both sex and tissue type, pointing to a context-dependent response to β-alanine depletion.

**Figure 5:**
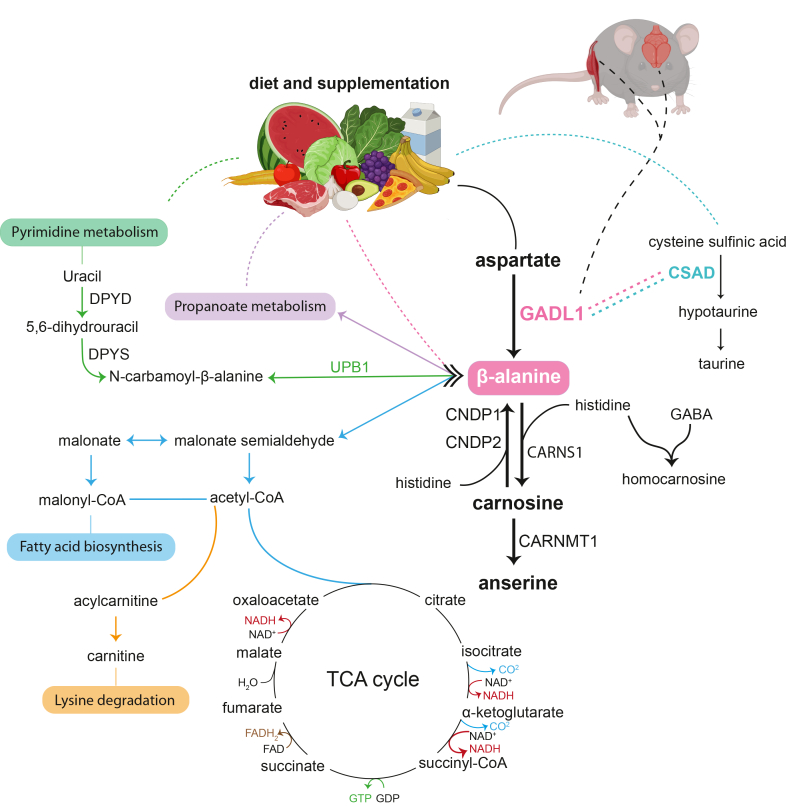
**Sources and fates of β-alanine metabolism with emphasis on GADL1**. GADL1 (pink) catalyzes the decarboxylation of aspartate to β-alanine, a precursor of HCDs such as carnosine and anserine. It is highly expressed in the olfactory bulb, cerebrum, and skeletal muscle, sharing 59 % sequence homology with CSAD (teal) and partial functional overlap with CSAD in taurine biosynthesis. In mammals, the main endogenous source of β-alanine is pyrimidine metabolism (green), catalyzed in its final step by UPB1. Additional contributors include dietary intake, pantothenate/CoA degradation (light pink), propanoate metabolism (purple), fatty acid biosynthesis (blue), lysine degradation (yellow), and intermediates of the TCA cycle. Created with BioRender and Adobe Illustrator.

To our knowledge, the *Gadl1* and *Carns1* mouse models are the only systems available to study endogenous carnosine synthesis in animals. A comparison with CARNS1 KO mice shows that loss of GADL1 produces widespread metabolic effects extending beyond carnosine depletion. Unlike *Gadl1*^*−/−*^ mice, *Carns1*^*−/−*^ mice do not display increased oxidative stress [53], suggesting that β-alanine deficiency, and possibly other metabolic alterations in *Gadl1*^*−/−*^ mice, contribute to a redox imbalance. Several dietary sources and metabolic sources contribute to β-alanine availability, including oral intake of β-alanine or its peptide derivatives, and *de novo* synthesis from aspartate and uracil. In animal tissues, β-alanine production from uracil involves a three-step enzymatic pathway: 1) dihydropyrimidine dehydrogenase (DPYD) catalyzes the reduction of uracil to dihydrouracil, 2) dihydropyrimidinase (DHP or DPYS) hydrolyzes dihydrouracil to N-carbamyl-β-alanine, and 3) β-Ureidopropionase (UPB1) converts N-carbamyl-β-alanine to β-alanine [54] (Figure 5). Autosomal recessive disorders due to mutations have been described in all three enzymes (OMIM 274270, 222748, 613161). Loss-of-function mutations in *UPB1* have been reported to result in severe neurodevelopmental deficiencies. However, more recent data suggest that many mutation carriers are asymptomatic, and that β-alanine and carnosine levels may remain within the normal range [55], leading to *UPB1* being classified as a 'gene of uncertain clinical significance’. Together, these findings indicate that GADL1-catalyzed decarboxylation of Asp contributes substantially to β-alanine synthesis in mammalian tissues and that blockade of this pathway has widespread metabolic consequences (Fig. 5).

GADL1 shares approximately 61 % amino acid sequence identity with CSAD, the rate-limiting enzyme in taurine synthesis [12,13]. Based on this structural homology and *in vitro* enzyme assays, it has been proposed that GADL1 may contribute to the biosynthesis of hypotaurine and taurine in certain tissues or species [11,12]. In the present study, however, no significant alterations in taurine or related metabolites were observed in the brain of *Gadl1*^*−/−*^ mice, consistent with our previous findings [11]. Notably, female mice showed a moderate increase in CSAD expression, suggesting compensatory regulation, although this requires further investigation. Importantly, without additional experimental evidence, it cannot be concluded that the observed changes are exclusively driven by β-alanine or its downstream metabolites. Taurine also plays key roles in calcium transport and homeostasis [56] and contributes to neural signaling in the OB [57]. Thus, some changes may involve taurine-dependent processes or other unidentified functions of GADL1.

While this study provides a comprehensive molecular characterization of *Gadl1*^*−/−*^ mice across transcriptomic, metabolomic, and proteomic datasets, several limitations should be noted. Sample sizes ranged from 4 to 7 per group, which may limit statistical power and sensitivity to detect subtle molecular effects. All findings are based on high-throughput datasets without targeted experimental validation. However, in our initial characterization of this mouse model, we validated key metabolites using NMR spectroscopy and targeted HPLC assays, RNA transcripts using quantitative reverse transcription PCR, and protein levels using western blotting [11]. While pathway- and category-level analyses support the biological relevance of the observed changes, additional targeted experiments will be required to validate specific molecular effects of *Gadl1* deletion. Together, these findings suggest that the molecular effects of GADL1 loss are partially buffered at the functional level.

Despite pronounced molecular alterations in the olfactory bulb, these changes did not translate into measurable deficits in olfactory-guided behavior. In a preliminary buried food-seeking test (Supplementary Fig. a6), *Gadl1*^*−/−*^ mice performed similarly to *Gadl1*^*+/+*^ controls, albeit with considerable interindividual variation, suggesting that GADL1 is not essential for olfaction, or that molecular remodeling preserves functional output. This type of functional compensation is common in genetic knockout models, where metabolic and cellular networks adapt to preserve system-level output despite underlying molecular perturbations. In brain tissues, HCDs [38] and GADL1 are particularly enriched in oligodendrocytes [11,13]. Oligodendrocyte and neuronal metabolism are highly integrated and essential for preserving neuronal integrity, for example, by preventing oxidative damage and providing a metabolic reserve to protect axons under glucose deprivation through lipid catabolism and fatty acid β-oxidation [58]. Altered oligodendrocyte metabolism has also been implicated in neurodegenerative diseases, including Alzheimer's disease and amyotrophic lateral sclerosis (ALS) [59,60]. Interestingly, GADL1 expression in oligodendrocytes is increased in the SOD1 mouse model of ALS [14], highlighting its potential neuroprotective role. While the observed changes in lipid and energy metabolism may reflect oligodendrocyte-specific alterations, cell-specific analyses and developmentally targeted conditional deletion of *Gadl1* are needed to fully understand the specificity of these changes. Finally, metabolomic measurements of the cerebrum were performed in male and female mice at different ages (12 vs 19–22 weeks). Although all animals were fully mature and genotype comparisons were performed within sex-matched cohorts, potential age-related influences cannot be excluded for cross-sex comparisons, which should therefore be interpreted cautiously. Future studies using larger age-matched cohorts of both sexes, targeted validation, and conditional knockouts could improve the mechanistic understanding of GADL1-dependent metabolism.

## 5 Conclusion

Our findings establish that the primary function of GADL1 is the decarboxylation of aspartic acid to β-alanine. Depletion of β-alanine seems to trigger a cascade of secondary metabolic alterations with distinct sex- and tissue-dependent patterns, possibly driven by compensatory regulation of metabolite levels. Female mice exhibit broader compensatory responses, whereas male mice show more localized but functionally relevant changes, particularly in the olfactory bulb. The *Gadl1* mouse model provides valuable insight into the consequences of GADL1 loss and highlights the potential of β-alanine supplementation to modulate metabolic functions, extending beyond its established role in enhancing athletic performance.

## CRediT authorship contribution statement

**Selina Cannon Homaei:** Writing – review & editing, Writing – original draft, Visualization, Validation, Project administration, Methodology, Investigation, Formal analysis, Data curation, Conceptualization. **Elaheh Mahootchi:** Writing – review & editing, Conceptualization. **Aashish Srivastava:** Writing – review & editing, Validation, Methodology, Formal analysis, Data curation. **Mahima Sanjay Gomladu:** Writing – review & editing, Validation, Methodology, Formal analysis, Data curation. **Oda Caspara Krokengen:** Writing – review & editing, Validation, Investigation. **Anne Baumann:** Writing – review & editing, Investigation, Conceptualization. **Jan Haavik:** Writing – review & editing, Supervision, Resources, Methodology, Funding acquisition, Conceptualization.

## Funding

This work was supported by 10.13039/100007793Stiftelsen Kristian Gerhard Jebsen (SKJ-MED-02), the Research Council of Norway (10.13039/100018724RCN) project 331725, Regional 10.13039/100018696Health Authority of Western Norway (no. 25048), the 10.13039/501100010767Innovative Medicines Initiative 2 Joint Undertaking (no. 115916 (PRISM: EU 10.13039/501100007601Horizon 2020, and 10.13039/100013322EFPIA), with RNA sequencing performed by the Genomics Core Facility (GCF), 10.13039/501100005036University of Bergen (10.13039/100018724RCN Project 245979/F50; Trond Mohn stiftelsen BFS2017TMT04, BFS2017TMT08) and proteomics by the Proteomics Unit at the 10.13039/501100005036University of Bergen (PROBE) which is a member of the National Network of Advanced Proteomics Infrastructure (NAPI); funded by the 10.13039/100018724RCN (INFRASTRUKTUR-programproject number: 295910).

## Declaration of competing interest

The authors declare that they have no conflict of interest.

## Data Availability

Data will be made available on request.
