# Supplementary material for: Blocking β-alanine synthesis triggers widespread perturbations of energy and lipid metabolism in the brain
Source: Mol Metab. 2026 May 23;109:102383. doi: 10.1016/j.molmet.2026.102383 (PMC13265694; doi:10.1016/j.molmet.2026.102383)
Supplement: Multimedia component 1 [file mmc1.docx]

**Appendix A Supplementary Material**

Supplementary Table a1A). **Overview of samples used in metabolomic analyses (heatmaps, dotplots, enrichment).**

|  | Olfactory bulb | | | | Cerebrum | | | | Cerebellum | |
| --- | --- | --- | --- | --- | --- | --- | --- | --- | --- | --- |
|  | **Female** | | **Male** | | **Female** | | **Male** | | **Male** | |
| **Age-range (weeks)** | 3-5 | | 3-7 | | 19-22 | | 12 | | 12 | |
| **Genotype** | WT | KO | WT | KO | WT | KO | WT | KO | WT | KO |
| **Number of mice** | 4 | 4 | 4 | 4 | 7 | 7 | 5 | 5 | 5 | 5 |
| **Total** | 8 | | 8 | | 14 | | 10 | | 10 | |

Supplementary Table a1B). **Overview of samples used in random-effects meta-analyses, in addition to samples from Table 1A.**

|  | Cerebrum and plasma | | Kidney | | Liver | | | | SKM | | | | | | | |
| --- | --- | --- | --- | --- | --- | --- | --- | --- | --- | --- | --- | --- | --- | --- | --- | --- |
|  | **Male** | | **Male** | | **Female** | | **Male** | | **Female** | | | | **Male** | | | |
| **Age-range (weeks)** | 3-7 | | 12 | | 19-22 | | 3-7 | | 3-5 | | 19-22 | | 3-7 | | 12 | |
| **Genotype** | WT | KO | WT | KO | WT | KO | WT | KO | WT | KO | WT | KO | WT | KO | WT | KO |
| **Number of mice** | 4 | 4 | 5 | 5 | 7 | 7 | 4 | 4 | 4 | 4 | 7 | 7 | 4 | 4 | 5 | 5 |
| **Total** | 10 | | 10 | | 14 | | 8 | | 8 | | 14 | | 8 | | 10 | |

Supplementary Table a2. **Overview of samples used in mRNA sequencing**

| **Tissue** | **Cerebrum** | | | | **Olfactory bulb** | | | |
| --- | --- | --- | --- | --- | --- | --- | --- | --- |
| **Sex** | Male | | Female | | Male | | Female | |
| **Genotype** | WT | KO | WT | KO | WT | KO | WT | KO |
| **Number of mice** | 5 | 5 | 5 | 5 | 4 | 6 | 4 | 4 |
| **Total** | 10 | | 10 | | 10 | | 8 | |

Supplementary Table a3. **Random-effects meta-analyses of mono- and diacylglycerols.** Significant results are marked in green.

|  |  |  | **Monoacylglycerols** | | | **Diacylglycerols** | | |
| --- | --- | --- | --- | --- | --- | --- | --- | --- |
| **Sex** | **Age group** | **Tissue** | **Pooled effect** | **I^2^** | **P-value** | **Pooled effect** | **I^2^** | **P-value** |
| Male | Young  (<12 weeks) | Olfactory bulb | Na | 0 % | 0.213 | 5.9 % | 0 % | 0.420 |
|  |  | Cerebrum | - 30.2 % | 0 % | 7.82e-05 | 6.1 % | 0 % | 0.140 |
|  |  | Liver | - 55.5 % | 0 % | 3.30e-09 | - 10.6 % | 0 % | 0.003 |
|  |  | Plasma | 61.5 % | 0 % | 0.002 | 2.9 % | 0 % | 0.488 |
|  |  | Skeletal muscle | 21.8 % | 0 % | 0.011 | 1.7 % | 0 % | 0.039 |
|  | Mature adult  (>12 weeks) | Cerebrum | 1.6 % | 0 % | 0.837 | 6.1 % | 0 % | 0.140 |
|  |  | Cerebellum | 32.0 % | 0 % | 0.010 | 11.9 % | 0.6 % | 8.55e-06 |
|  |  | Kidney | - 4.1 % | 0 % | 0.360 | 10.3 % | 14.9 % | 8.05e-05 |
|  |  | Liver | 3.9 % | 13.6 % | 0.299 | 22.8 % | 40.2 % | 3.16e-10 |
|  |  | Skeletal muscle | - 0.3 % | 0 % | 0.954 | 101.2 % | 0 % | 1.73e-84 |
| Female | Young  (<12 weeks) | Olfactory bulb | 0 % | 22.7 % | 0.986 | 119.2 % | 68.6 % | 1.24e-19 |
|  |  | Skeletal muscle | - 15.5 % | 0 % | 0.025 | - 52.9 % | 0 % | 2.29e-11 |
|  | Mature adult  (>12 weeks) | Cerebrum | - 0.43 % | 0 % | 1.11e-05 | 0 % | 0 % | 0.894 |
|  |  | Liver | 4.3 % | 0 % | 0.374 | 4.3 % | 0 % | 0.016 |
|  |  | Skeletal muscle | - 34.9 % | 29.4 % | 2.12e-12 | - 17.6 % | 0 % | 7.95e-06 |

Supplementary Fig. a1. **Global variance across tissues and omics layers.** Principal Component Analysis (PCA) of (A, C) metabolomic, (B, D) transcriptomic, and (E) proteomic profiles. In the olfactory bulb (A-B), samples display clear genotype-driven separation along PC1, whereas cerebrum samples (C-E) show greater homeostatic stability with overlapping clusters. Across all datasets, sex consistently emerges as a secondary contributor to variance (PC2).


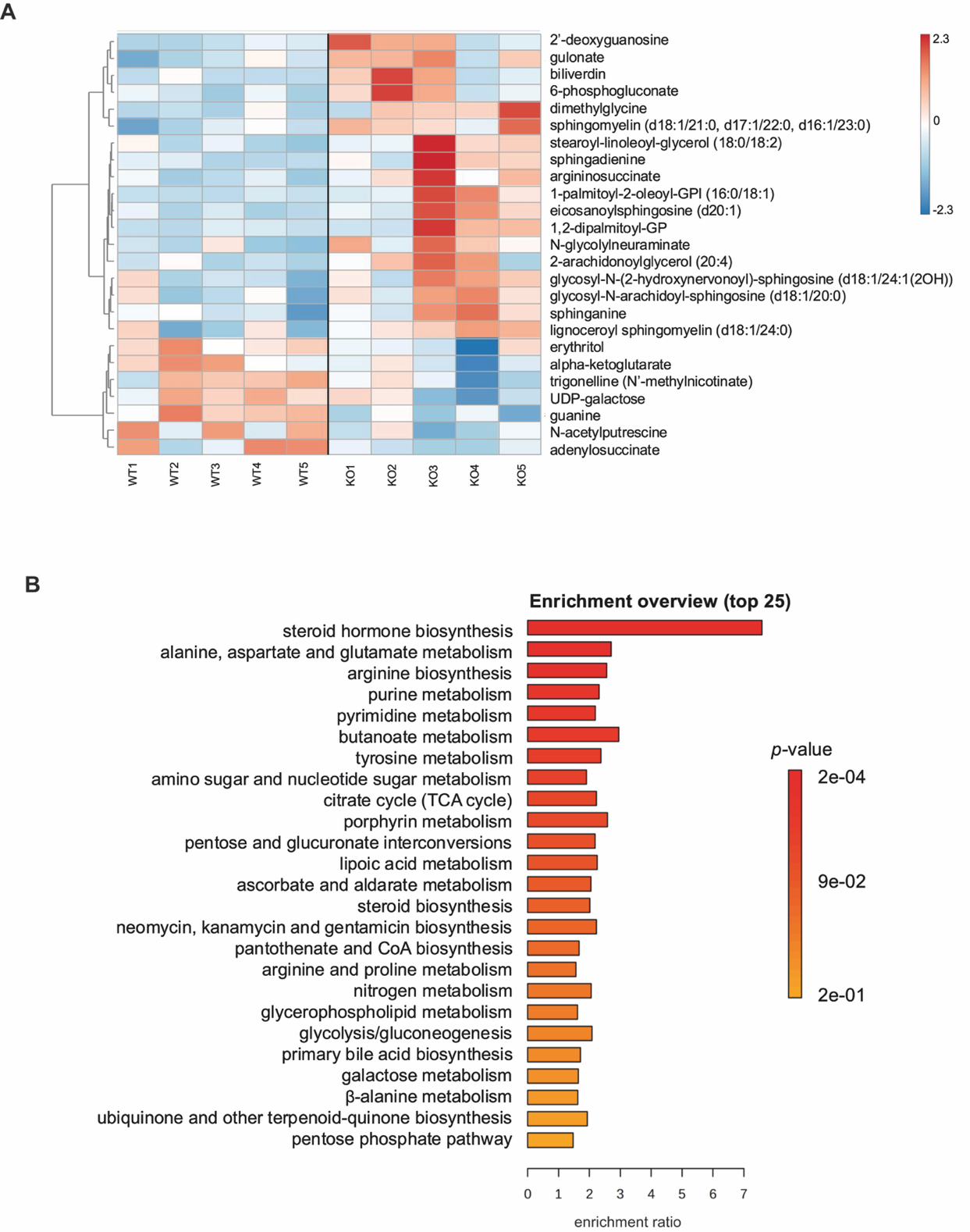


Supplementary Fig. a2. **Metabolomic profiling of cerebrum tissue.** (A) Hierarchical heatmap and (B) MSEA results from male cerebellum tissue.

**
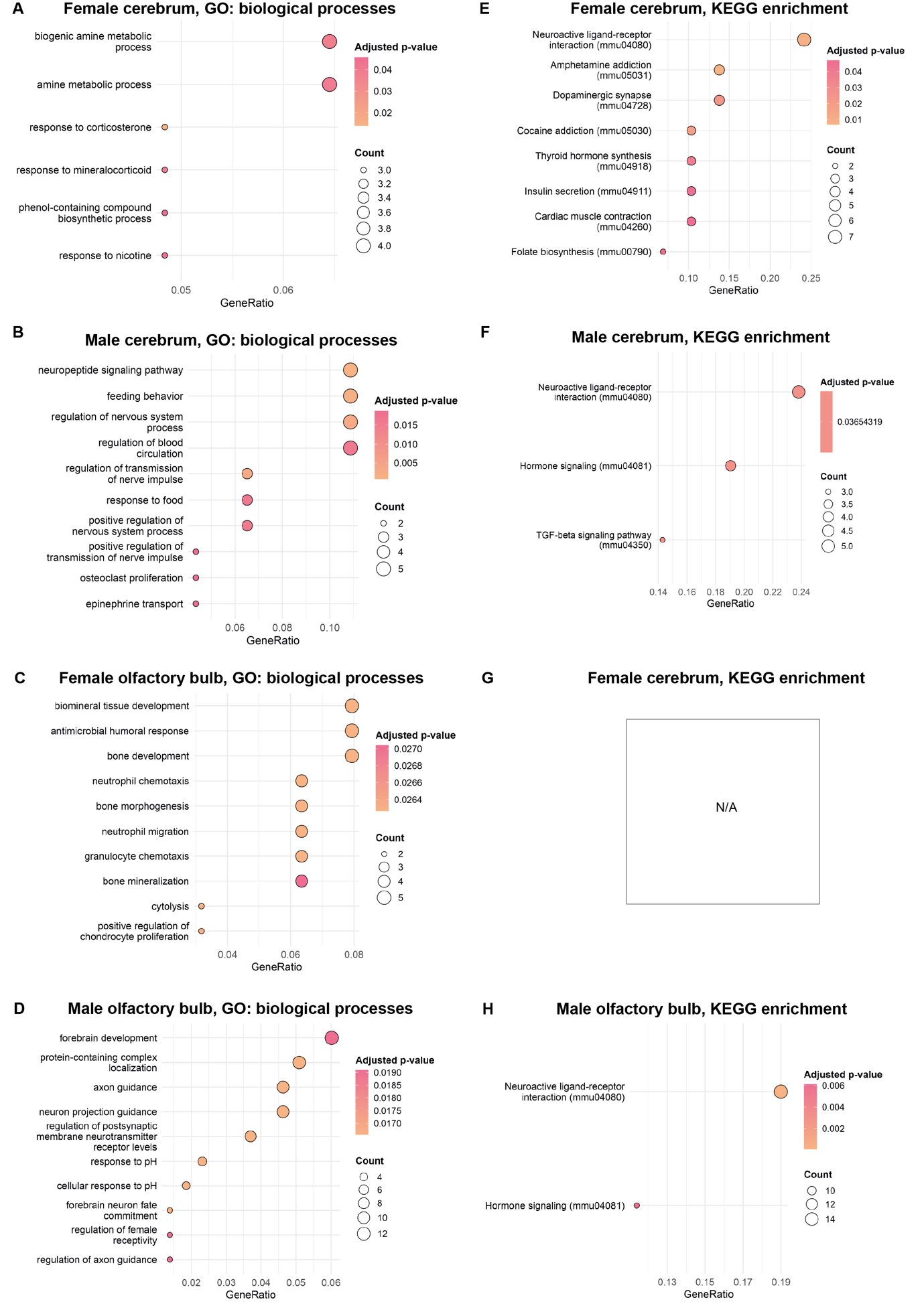
**

Supplementary Fig. a3. **Functional enrichment analysis of differentially expressed genes from mRNA sequencing data.** (A) Female and (B) male cerebrum, and (C) female and (D) male olfactory bulb.

Supplementary Fig. a4. **Integrative multi-omics network analysis.** Functional interaction networks for female (A, C) and male (B, D) tissues. (A) The female olfactory bulb network reveals a metabolic hub centered on Plb1, connecting major phospholipid alterations, while (B) the male olfactory bulb network highlights shifts in cytoskeletal and contractile components (e.g., *Myh3, Mylk3*). (C) Female cerebrum integration shows coordinated fatty acid and neurotransmitter regulation, whereas (D) male cerebrum integration is characterized by ribosomal and mitochondrial translation. Pink nodes represent proteins/genes, and blue nodes represent metabolites.

Supplementary Fig. a5. **Schematic overview of sample distribution and significantly altered features across omics layers.** (A) Distribution of analyzed samples; (B) significantly altered features across omics layers (p < 0.05); VENN diagrams of significantly altered features in (C) transcriptomics, (D) proteomics, and (E) metabolomics for male (orange) and female (pink) cerebrum, and male (green) and female (blue) olfactory bulb.


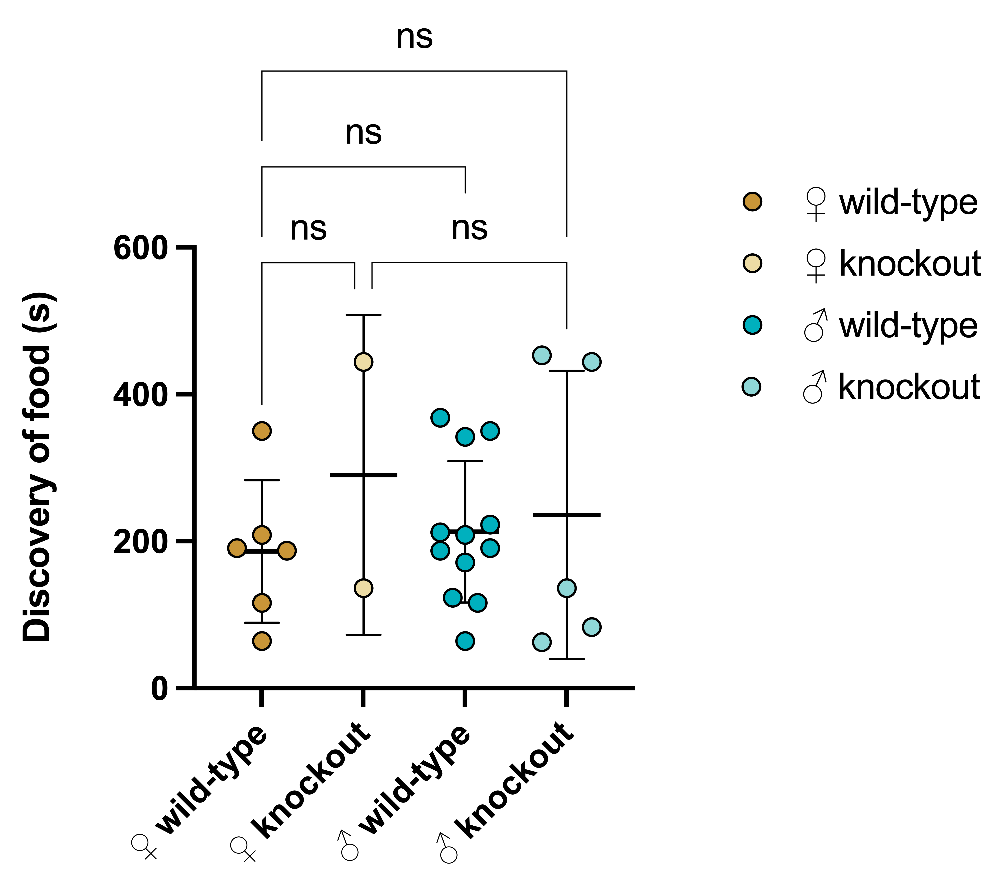


Supplementary Fig. a6. **Buried food-seeking test.** The experiment followed the protocol of Machado et al. [1]. Mice were food-deprived for 24 hours prior to testing. Each animal was placed in a clean cage with fresh bedding, in which a 2 g food pellet was buried. Motivation was assessed on a separate day using an unburied food-seeking test, as described by Li et. al. [2], with the pellet placed on top of the bedding. All video recordings were scored by an independent investigator blinded to group assignments. No significant differences were observed, although biological variability and the limited sample size may have influenced the results.

1. Machado CF, Reis-Silva TM, Lyra CS, Felicio LF and Malnic B (2018) Buried Food-seeking Test for the Assessment of Olfactory Detection in Mice. Bio Protoc 8:e2897. doi: 10.21769/BioProtoc.2897

2. Li F, Ponissery-Saidu S, Yee KK, Wang H, Chen ML, Iguchi N, Zhang G, Jiang P, Reisert J and Huang L (2013) Heterotrimeric G protein subunit Ggamma13 is critical to olfaction. J Neurosci 33:7975-84. doi: 10.1523/JNEUROSCI.5563-12.2013
